# Bilateral cavo-ilio-femoral thrombosis in an adolescent with transient anti-phospholipid antibodies and Factor V heterozygous mutation: a case report

**DOI:** 10.4076/1757-1626-2-6830

**Published:** 2009-08-05

**Authors:** Giulio Frontino, Arianna Passoni, Maria Antonietta Piscopo, Elena Grechi, Bruna Cammarata, Gabriella Pozzobon

**Affiliations:** Department of Pediatrics, San Raffaele Scientific InstituteVia Olgettina 60, 20132, MilanItaly

## Abstract

We report a case of bilateral cavo-ilio-femoral thrombosis in an adolescent with factor V heterozygous mutation and transient antiphospholipid antibodies secondary Varicella infection.

The clinical significance of finding transient antiphospholipid antibodies in the sera of infectious disease is unclear. We here report a case of bilateral cavo-ilio-femoral thrombosis in an adolescent with newly diagnosed factor V heterozygous mutation and transient antiphospholipid antibodies secondary to Varicella infection.

## Introduction

Factor V Leiden is the most common cause of inherited thrombophilia, accounting for 40 to 80% of cases. The prothrombin gene mutation, deficiencies in protein S, protein C, and antithrombin account for most of the remaining cases [1,2]. The total incidence of an inherited thrombophilia in subjects with a deep vein thrombosis ranges from 24 to 37% overall, compared to about 10 percent in controls. Heterozygosity for the factor V Leiden mutation accounts for 90 to 95% of cases of the activated protein C (APC) resistance phenotype. A much smaller number of homozygotes exist. Acquired conditions that can influence first generation APC resistance assays include elevated factor VIII levels, pregnancy, use of oral contraceptive, and the presence of antiphospholipid antibodies. The prevalence of heterozygosity for the factor V Leiden mutation in Caucasians, including Europeans, Jewish, Israeli, Arab, Canadian, and Indian populations, ranges from 1 to 8.5%.

Antiphospholipid syndrome (APS) is a systemic autoimmune disorder characterized by a combination of arterial or venous thrombosis and recurrent fetal loss, accompanied by elevated titers of antiphospholipid antibodies (aPL), namely the lupus anticoagulant (LAC) and anticardiolipin antibodies (aCL) [3,4]. Also the anti beta2-GPI is among the most commonly detected subgroups of aPL antibodies. The syndrome may occur in isolation (primary APS) or in association with an underlying systemic disease, particularly systemic lupus erythematosus (SLE) (secondary APS). Since the association between antiphospholipid antibodies (aPL) and syphilis was first described, many other viral, bacterial and parasitic infections have been shown to induce antiphospholipid antibodies, notably anticardiolipin antibodies (aCL). A review of the literature shows that while aCL occur frequently in viral infections, particularly in HIV (49.75%), HBV (24%) and HCV (20%), it is very rarely associated with anti-β2 glycoprotein I antibodies (anti-β2GPI) and is not correlated with thrombosis risk or hematological manifestations of APS. Concerning bacterial infections, aCL is often present in leprosy (42.7%), where it is frequently associated with the presence of anti-β2GPI (44.8%), and in syphilis infections (8 to 67%), though without correlation with thrombotic events [5].

## Case presentation

We report a case of 16-year-old male from Romania with bilateral cavo-ilio-femoral thrombosis who recently recovered from Varicella infection. The patient had not suffered lack of mobility during the acute viral illness.

Several days after recovery from the infection, the patient reached the emergency department referring strangury, an increasing lumbar pain irradiating to the left leg, and claudication. An abdominal Doppler ultrasound was performed which demonstrated complete thrombosis of the inferior vena cava up to the renal veins, with involvement of the iliac and femoral veins bilaterally. Chest and abdominal CT scans confirmed the bilateral cavo-ilio-femoral thrombosis and excluded the presence of pulmonary thromboemboli. While awaiting laboratory results for thromphylic screening, anticoagulant therapy was started with progressive physical recovery. The thrombophylic screening (including homocysteine, lupus-like anticoagulants, Protein C, Protein S, plasminogen, IgG and IgM anticardiolipin and anti-β2-glycoprotein I) demonstrated the presence of IgM anti-cardiolipin antibodies (30.5 MPL), IgM anti-β2-glycoprotein I antibodies (60 U/ml), and heterozygosis for mutation of the Factor Leiden gene. Three months later the aPL antibodies were no longer detectable.

## Discussion

The appearance of antiphospholipid antibodies during viral infections (especially parvovirus B19, cytomegalovirus, *Varicella zoster* virus, and HIV) is well documented and is generally considered transient and not associated to the progression towards an APS. However, the development of thrombotic events during important viral and bacterial infections is not infrequent.

Mutations in the Factor V gene, along with those in the prothrombin gene, are the most common causes of hereditary thrombophilia. The risk of thrombotic events shows an 80 fold increase in subjects affected homozygote mutations in Factor V when compared to that of healthy subjects. The risk is 7 fold higher in those subjects affected by heterozygote mutations. As a result, only about 10% of subjects with the heterozygote mutation develops at least on thrombotic event in his or her lifetime.

Several cases of thrombosis post-Varicella infection have been reported, but only one case of ilio-femoral thrombosis post-Varicella infection in a patient with factor V mutation is present in literature [5]. Uthman et al reported the case of a 16-year-old boy who developed APS 1 week after the occurrence of chickenpox [6]. He presented with acute right ilio-femoro-popliteal deep vein thrombosis (DVT) [7]. IgM and IgG aCL antibodies were both positive on admission. IgM aCL antibodies remained positive 6 weeks later, which suggests their role as a predisposing factor for DVT. Manco-Johnson et al [8], in a study investigating the cause of purpura fulminans, disseminated intravascular coagulation, or thrombosis in 7 children with Varicella, reported the association of thrombosis with the presence of aPL antibodies in Varicella infections. Moreover, the investigators found all the children to have a LAC and acquired protein S deficiency. In addition, 4 of the patients had aPL or aCL antibodies. Peyton et al [9] also reported the cases of 2 men with Varicella pneumonia who had profound lower extremity ischemia caused by thrombosis of the profunda femoris and tibial arteries. Both patients had free protein S deficiency. IgG and IgM aPL antibodies were present in one, whereas the other had evidence of the LAC. In addition, Barcat et al [10] reported a case of DVT in an adult with Varicella. Although there was no evidence of thrombophilia, a transient significant level of aPL antibodies and LAC were observed.

## Conclusions

In conclusion, the clinical significance of finding transient aCL and anti-β2-GPI antibodies in the sera of infectious disease remains unknown. In some patients, these aPL anti-β2-GPI antibodies are transient and disappear within 2 or 3 months. In some susceptible individuals, they raise the question of whether infections may be a trigger for the development of aPL and anti-β2-GPI antibodies which may consequently activate a cascade of prothrombotic events [10]. In our case, it is thus possible that the aPL antibodies developed during the *Varicella*-infection may have acted as a prothrombotic trigger in a case of pre-existing hereditary thrombophilia which may have otherwise remained undiagnosed. Consequently, greater caution is warranted in the monitoring of patients with inherited thrombophilia during infection, in order to prevent possible significant and life-threatening thrombotic complications.

## Abbreviations

aCL: anticardiolipin antibodies
anti-B2-GPI: anti-B2-glycoprotein I
aPL: antiphospholipid antibodies
APC: activated protein C
APS: Antiphospholipid Syndrome
DVT: deep vein thrombosis
LAC: Lupus anticoagulant
SLE: Sstemic Lupus Erythematosus
